# 660. Effect of the COVID-19 Pandemic on Blood Culture Contamination Rates and Quality Improvement Processes

**DOI:** 10.1093/ofid/ofab466.857

**Published:** 2021-12-04

**Authors:** Alexander G Hosse

**Affiliations:** Louisiana State University School of Medicine, Baton Rouge, Louisiana

## Abstract

**Background:**

Blood cultures are the gold standard for diagnosing bloodstream infections and a vital part of the work-up in systemic infections. However, contamination of blood cultures represents a significant burden on patients and the healthcare system with increased hospital length of stay, unnecessary antibiotics, and financial cost. The data discussed here offer insight into blood culture contamination rates before and through the COVID-19 pandemic at a community hospital and the processes that were affected by the pandemic.

**Methods:**

Blood culture contaminations were determined by using the number of sets of blood cultures with growth and the presence of an organism from the National Healthcare Safety Network's (NHSN) commensal organism. Contamination rates were evaluated by status as a standard unit or a COVID-19 isolation unit in either the emergency department (ED) or inpatient floor units. The identified four groups had different processes for drawing blood cultures, particularly in terms of training of staff in use of diversion devices. The electronic medical record was used to track contaminations and the use of diversion devices in the different units.

**Results:**

The inpatient COVID units were consistently elevated above the other units and the institutional contaminant goal of 2.25%, ranging from 9.6% to 13.3% from 4/2020-9/2020. Those units were the primary driver of the increase in overall contamination rates. COVID ED nursing staff (that had previously undergone training in the use of diversion devices) used diversion devices to draw 51 of 133 (38.3%) cultures compared to only 15 of 84 (17.9%) on the COVID inpatient units.

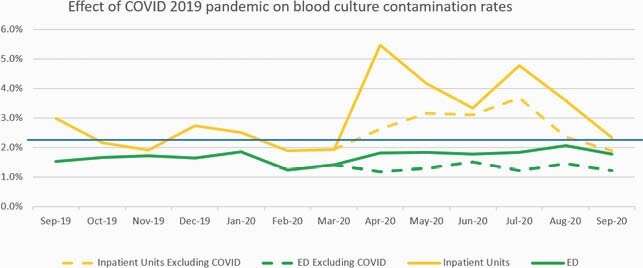

Figure 1. Comparison of contamination rates in the ED vs the inpatient units from all campuses from September 2019 through September 2020. The blue line represents the hospital goal of 2.25% contamination rate. Solid lines represent total contamination rates including COVID isolation units whereas dotted lines represent units excluding COVID isolation units.

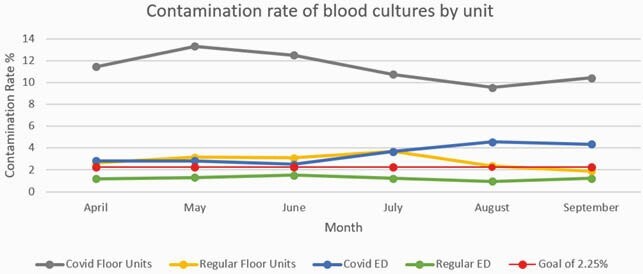

Figure 2. Comparison of the non-COVID vs COVID isolation units in the emergency department and inpatient units. The red line represents the hospital goal of less than 2.25% for blood culture contamination rate.

Table of Contaminants vs. Total Collected Blood Cultures in Each Unit by Month

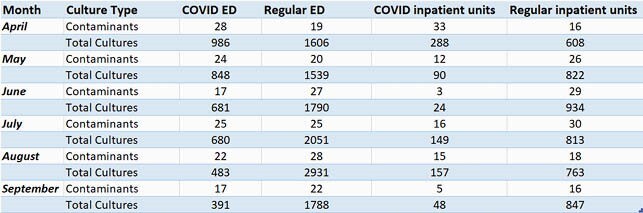

Figure 3. Raw data from Figure 2. Total blood culture contaminations from each unit by month compared to total blood culture collections from each unit by month.

**Conclusion:**

Evaluation revealed that nursing staff with less training in blood culture collection, particularly the use of diversion devices, were the primary staff collecting blood cultures in the inpatient COVID units. The difference in training is felt to be the primary driver of the increase in contaminants in the inpatient COVID units. The marked increase in contaminations highlights the difficulties of maintaining quality control processes during an evolving pandemic and the importance of ongoing efforts to improve the quality of care. These findings demonstrate the importance of training and routine use of procedures to reduce contaminations even during.

**Disclosures:**

**All Authors**: No reported disclosures

